# Supplementary material for: Modeling glioblastoma heterogeneity as a dynamic network of cell states
Source: Mol Syst Biol. 2021 Sep 16;17(9):e10105. doi: 10.15252/msb.202010105 (PMC8444284; doi:10.15252/msb.202010105)
Supplement: Supplementary file 5 — Source Data for Figure 3 [file MSB-17-e10105-s001.zip › Figure3A_sourcedata/GSEA_3065/hallmarks_state6.GseaPreranked.1623416308702/HALLMARK_ALLOGRAFT_REJECTION.html]

Details for gene set HALLMARK\_ALLOGRAFT\_REJECTION[GSEA]

|  || Dataset | state6 |
| Phenotype | NoPhenotypeAvailable |
| Upregulated in class | na\_pos |
| GeneSet | HALLMARK\_ALLOGRAFT\_REJECTION |
| Enrichment Score (ES) | 0.30329347 |
| Normalized Enrichment Score (NES) | 1.166309 |
| Nominal p-value | 0.20812182 |
| FDR q-value | 0.31923804 |
| FWER p-Value | 0.977 |
Table: GSEA Results Summary

  

Fig 1: Enrichment plot: HALLMARK\_ALLOGRAFT\_REJECTION      
 Profile of the Running ES Score & Positions of GeneSet Members on the Rank Ordered List

  

| PROBE | GENE SYMBOL | GENE\_TITLE | RANK IN GENE LIST | RANK METRIC SCORE | RUNNING ES | CORE ENRICHMENT || 1 | RPS19 |  |  | 14 | 0.295 | 0.0562 | Yes |
| 2 | CCND2 |  |  | 32 | 0.269 | 0.1068 | Yes |
| 3 | RPS3A |  |  | 80 | 0.224 | 0.1453 | Yes |
| 4 | RPL39 |  |  | 82 | 0.223 | 0.1889 | Yes |
| 5 | RPS9 |  |  | 230 | 0.166 | 0.2046 | Yes |
| 6 | RPL9 |  |  | 290 | 0.153 | 0.2277 | Yes |
| 7 | EIF5A |  |  | 573 | 0.116 | 0.2182 | Yes |
| 8 | HDAC9 |  |  | 574 | 0.116 | 0.2409 | Yes |
| 9 | BCL10 |  |  | 588 | 0.115 | 0.2620 | Yes |
| 10 | MAP3K7 |  |  | 694 | 0.106 | 0.2707 | Yes |
| 11 | APBB1 |  |  | 758 | 0.102 | 0.2834 | Yes |
| 12 | BCAT1 |  |  | 759 | 0.102 | 0.3033 | Yes |
| 13 | UBE2N |  |  | 947 | 0.089 | 0.2992 | No |
| 14 | CSK |  |  | 1055 | 0.082 | 0.3031 | No |
| 15 | ABI1 |  |  | 1465 | 0.066 | 0.2691 | No |
| 16 | TPD52 |  |  | 1899 | 0.051 | 0.2295 | No |
| 17 | EIF4G3 |  |  | 1963 | 0.049 | 0.2318 | No |
| 18 | ACVR2A |  |  | 2368 | 0.037 | 0.1928 | No |
| 19 | STAT1 |  |  | 2471 | 0.034 | 0.1878 | No |
| 20 | MTIF2 |  |  | 2743 | 0.028 | 0.1623 | No |
| 21 | EIF3J |  |  | 2953 | 0.023 | 0.1428 | No |
| 22 | ABCE1 |  |  | 3395 | 0.013 | 0.0949 | No |
| 23 | IRF7 |  |  | 3602 | 0.009 | 0.0732 | No |
| 24 | EIF3D |  |  | 3862 | 0.003 | 0.0442 | No |
| 25 | HIF1A |  |  | 3882 | 0.003 | 0.0426 | No |
| 26 | CCND3 |  |  | 4016 | 0.000 | 0.0274 | No |
| 27 | CSF1 |  |  | 4350 | -0.006 | -0.0095 | No |
| 28 | TAPBP |  |  | 4513 | -0.009 | -0.0263 | No |
| 29 | IL11 |  |  | 4790 | -0.014 | -0.0552 | No |
| 30 | CAPG |  |  | 4878 | -0.016 | -0.0620 | No |
| 31 | IKBKB |  |  | 4921 | -0.017 | -0.0635 | No |
| 32 | AKT1 |  |  | 5071 | -0.020 | -0.0767 | No |
| 33 | NCK1 |  |  | 5409 | -0.027 | -0.1100 | No |
| 34 | NPM1 |  |  | 5412 | -0.027 | -0.1051 | No |
| 35 | SOCS5 |  |  | 5419 | -0.027 | -0.1005 | No |
| 36 | IFNAR2 |  |  | 5455 | -0.028 | -0.0991 | No |
| 37 | NME1 |  |  | 5479 | -0.028 | -0.0963 | No |
| 38 | GALNT1 |  |  | 5567 | -0.030 | -0.1004 | No |
| 39 | F2R |  |  | 5881 | -0.036 | -0.1293 | No |
| 40 | UBE2D1 |  |  | 6196 | -0.043 | -0.1569 | No |
| 41 | RIPK2 |  |  | 6203 | -0.043 | -0.1492 | No |
| 42 | IL27RA |  |  | 6412 | -0.047 | -0.1637 | No |
| 43 | TGFB1 |  |  | 6712 | -0.056 | -0.1869 | No |
| 44 | CD74 |  |  | 6826 | -0.059 | -0.1882 | No |
| 45 | MRPL3 |  |  | 6871 | -0.061 | -0.1814 | No |
| 46 | IFNGR2 |  |  | 6997 | -0.065 | -0.1830 | No |
| 47 | EIF3A |  |  | 6999 | -0.065 | -0.1705 | No |
| 48 | TGFB2 |  |  | 7048 | -0.066 | -0.1630 | No |
| 49 | TAP2 |  |  | 7315 | -0.075 | -0.1788 | No |
| 50 | GLMN |  |  | 7512 | -0.082 | -0.1852 | No |
| 51 | DEGS1 |  |  | 7831 | -0.098 | -0.2025 | No |
| 52 | IFNGR1 |  |  | 7892 | -0.101 | -0.1896 | No |
| 53 | FLNA |  |  | 8171 | -0.119 | -0.1982 | No |
| 54 | EGFR |  |  | 8251 | -0.126 | -0.1826 | No |
| 55 | TAP1 |  |  | 8393 | -0.146 | -0.1702 | No |
| 56 | THY1 |  |  | 8454 | -0.153 | -0.1472 | No |
| 57 | CD47 |  |  | 8587 | -0.186 | -0.1260 | No |
| 58 | BRCA1 |  |  | 8659 | -0.217 | -0.0916 | No |
| 59 | B2M |  |  | 8718 | -0.258 | -0.0479 | No |
| 60 | TIMP1 |  |  | 8751 | -0.290 | 0.0052 | No |
Table: GSEA details [plain text format]

  

Fig 2: HALLMARK\_ALLOGRAFT\_REJECTION: Random ES distribution      
 Gene set null distribution of ES for **HALLMARK\_ALLOGRAFT\_REJECTION**
